# Disinfectant Activity of A Portable Ultraviolet C Equipment

**DOI:** 10.3390/ijerph16234747

**Published:** 2019-11-27

**Authors:** Andrea Guridi, Elena Sevillano, Iñigo de la Fuente, Estibaliz Mateo, Elena Eraso, Guillermo Quindós

**Affiliations:** 1 Department of Immunology, Microbiology and Parasitology, Faculty of Pharmacy, University of the Basque Country UPV/EHU, 01006 Vitoria-Gasteiz, Spain; andrea.guridi@ehu.eus; 2 UFI 11/25 «Microbios y Salud», Department of Immunology, Microbiology and Parasitology, Faculty of Medicine and Nursing, University of the Basque Country, UPV/EHU Apartado 699, 48080 Bilbao, Spain; idelafuente009@ikasle.ehu.eus (I.d.l.F.); estibaliz.mateo@ehu.eus (E.M.); elena.eraso@ehu.eus (E.E.); guillermo.quindos@ehu.eus (G.Q.)

**Keywords:** UVC light disinfection, portable equipment, healthcare-associated infections, disinfection

## Abstract

Healthcare-associated infections (HAIs) can be caused by microorganisms present in common practice instruments generating major health problems in the hospital environment. The aim of this work was to evaluate the disinfection capacity of a portable ultraviolet C equipment (UV Sanitizer Corvent^®^ -UVSC-) developed to disinfect different objects. For this purpose, six pathogens causing HAIs: *Acinetobacter baumannii*, *Bacillus subtilis*, *Escherichia coli*, *Pseudomonas aeruginosa*, *Staphylococcus aureus* and *Candida albicans*, were inoculated on slides and discs of different biomaterials (borosilicate, polycarbonate, polyurethane, silicone, Teflon and titanium) and exposed to ultraviolet C radiation. UVSC disinfection was compared with ethanol and chlorhexidine antimicrobial activities following the standards EN14561 and EN14562. Disinfection, established as a reduction of five logarithms from the initial inoculum, was achieved with the UVSC at 120 s of exposure time, with and without the presence of organic matter. The disinfectant effect was observed against *S. aureus*, *P. aeruginosa*, *E. coli*, *B. subtilis* and *C. albicans* (reduction >99.999%). Disinfection was also achieved with 70% ethanol and 2% chlorhexidine. As conclusion, UVSC was effective disinfecting the most contaminated surfaces assayed, being a promising alternative for disinfecting hospital materials and inanimate objects that cannot be immersed in liquid biocides, reducing the risk of pathogen transmission.

## 1. Introduction

Healthcare-associated infections (HAIs) prolong hospital stay, cause long-term disability and additional costs for health systems, patients and their families, and preventable deaths [1]. Furthermore, HAIs increase the possibility of selecting multidrug resistant microorganisms. Microorganisms causing HAIs belong to different groups, such as Gram-negative (*Acinetobacter baumannii*, *Pseudomonas aeruginosa* and *Escherichia coli*) and Gram-positive bacteria (*Staphylococcus aureus*), spore-producing bacteria (*Bacillus* spp.) and yeasts (*Candida albicans*) [1,2]. These pathogens can be spread by the hands of healthcare personnel and by patient-to-patient contact [3,4]. Some pathogens can persist in the hospital environment colonizing surfaces, such as doorknobs, faucets, electronic devices, hospital supplies, television remote controls or inanimate objects belonging to patients, such as mobile phones. All these inanimate surfaces hardly ever are disinfected and can act as fomites [5,6]. Besides, these objects can be used both inside and outside the hospital and other healthcare settings, and thus can contribute to increase the risk for pathogen spreading [7,8,9]. While medical equipment disinfection is a common practice, to establish hard surfaces disinfection measures could be of great importance for inanimate objects in contact with highly colonized areas of the patient, such as hands, mouth, nose and ears that could be potential sources of HAIs and community infections [7,9,10]. To avoid this microbial colonization and persistence on both fomites and hard surfaces, routine chemical and physical decontamination approaches have been introduced [4]. Ethanol and other alcohols, and biguanides like chlorhexidine, are widely used in hospitals and laboratories to disinfect surfaces and to prevent nosocomial infections [11,12]. However, not all the above-mentioned objects can be treated with chemical biocides due to possible deterioration of the material or the electronics [13,14].

Among physical methods or no touch technologies, ultraviolet C radiation (UVC) is widely used in disinfecting materials and hospitals wards, operating rooms and ICUs. DNA exposure to UVC inhibits cellular replication since it damages the cell by photohydration, photosplitting, photodimerization, and photocrosslinking [15]. Therefore, based on the UVC microbicide effect, a new portable, automated, easy to use and safe equipment, UV Sanitizer Corvent (UVSC), has been designed for disinfecting inanimate objects and devices in the hospital environment that could act like fomites, including sanitary materials such as phonendoscopes, thermometers, sphygmomanometer, otoscopes, etc. The aim of this study was to evaluate the capacity of UVSC to disinfect different materials contaminated with HAI-associated microorganisms in comparison to ethanol and chlorhexidine.

## 2. Materials and Methods

### 2.1. Study Design

Initially, the experiments were performed with the slides inoculated with the microorganisms and disinfection times specified according to the standards EN14561:2006 and EN14562:2006 (European Committee for standardization (CEN) 2006) [16,17]. These microorganisms were *S. aureus* CECT 435 and *P. aeruginosa* CECT 108 including clean and dirty conditions, and disinfection by using UVSC for 30 s, and by immersion in 96%, 70% and 60% ethanol or in 4%, 2% and 1% chlorhexidine for one hour. 

Secondly, the experiments were carried out with the slides inoculated with six microbial species causing HAIs, including clean and dirty conditions, and disinfected with UVSC, 70% ethanol and 2% chlorhexidine for 30, 60, 90 and 120 s.

Finally, the experiments were performed inoculating a Gram-negative bacterium (*P. aeruginosa* CECT 108), a Gram-positive bacterium (*S. aureus* CECT 435) and a yeast (*C. albicans* ATCC MYA-2876) on discs of six different materials, in both conditions. These discs were disinfected with UVSC, 70% ethanol and 2% chlorhexidine for 30, 60, 90 and 120 s.

### 2.2. Microbial Species and Materials Tested

Disinfection experiments were performed using six strains from de American Type Culture Collection (ATCC) (Rockville MD, USA) and the Spanish Type Culture Collection (CECT, Valencia, Spain): *Staphylococcus aureus* CECT 435, *Pseudomonas aeruginosa* CECT 108, *Escherichia coli* CECT 434, *Bacillus subtilis* ATCC 6051, *Acinetobacter baumannii* ATCC 19606 and *Candida albicans* ATCC MYA-2876™. Glassware slides and discs of borosilicate, polycarbonate, polyurethane, silicone, Teflon and titanium were used (Biosurface, Bozeman, MT, USA). These discs had 1.27 cm of diameter and 0.13 cm of thickness.

### 2.3. Disinfection Methods

**Figure 1:**
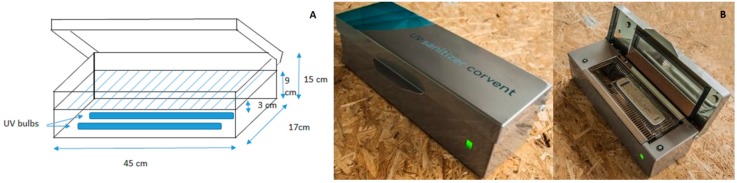
Corvent^®^ UV Sanitizer equipment. (**A**). Equipment layout. (**B**). Images of the equipment.

Three disinfection methods were used: UVSC, ethanol and chlorhexidine. UVSC (Corvent®, Matadepera, Spain. Utility model Spain number 201530274) is a stainless steel box of 17 cm wide, 15 cm high and 45 cm long that includes two ultraviolet bulbs placed at the bottom and a mirror on the cover. UVC radiation is emitted at a wavelength of 253.7 nm at 8 watts, with a delivered UVC dose value of 840 mJ/cm^2^, 1680 mJ/cm^2^, 2520 mJ/cm^2^ and 3360 mJ/cm^2^ in 30, 60, 90 and 120 s respectively and an irradiance of 28 mW/cm^2^ at the target surface. Before and after using the equipment it was switched on for 2 min to ensure its disinfection. Inanimate objects to be disinfected are placed inside a provided plastic bag (Sanitbag-SBC-, Corvent^®^, Matadepera, Spain) on a tray above the bulbs (at 3 cm distance) and when the equipment is tightly closed, UVC light bathes all sides of the object due to a mirror effect (Figure 1). The SBC is completely sealed after UVC bath ensuring that the object remains disinfected until use. Disinfection time specified by the manufacturer is 30 s. 

To compare the effectiveness of UVSC, slides and discs of different materials inoculated with different microorganisms were treated also with two common disinfectants, ethanol and chlorhexidine, according to the standards EN14561 and EN14562 that respectively describe the protocols for evaluating bactericidal, and fungicidal activities on instruments used in the medical area [16,17].

### 2.4. Microbial Inoculum Preparation

Assays were carried out under two different experimental conditions, in presence or absence of organic matter, according to above-mentioned EN14561 and EN14562 standards. Clean conditions, defined as the absence of organic matter on the surface, were performed by adding to the inoculum 0.3 g/L of bovine serum albumin as interfering substance. Dirty conditions, defined as the presence of organic matter, were tested by adding 3 g/L bovine serum albumin and 3 mL/L of sheep erythrocytes.

For preparing the inoculum, bacteria were grown on agar plates with trypticase soy agar (TSA) and *Candida* on Sabouraud dextrose agar (SDA), at 37 °C for 24–48 h. A test suspension of each microorganism was adjusted by spectrophotometry (OD600), and tested by culture to 1.5–5.0 × 10^9^ or 1.5–5.0 × 10^8^ colony-forming units (CFU) per mL for bacteria and *Candida*, respectively. Then, 0.9 mL of the inoculum were mixed with 0.1 mL of interfering substance for each condition, clean and dirty. A 50 μL volume of the mixture was inoculated in an area of 1 cm^2^ of slides or discs and left drying at room temperature (it took from 15 to 45 min to dry depending on the material). 

### 2.5. Assessment of Disinfectant Capacity on Glassware Slides

Initially, the experiments were performed on slides inoculated with *S. aureus* CECT 435 and *P. aeruginosa* CECT 108 [16,17] at clean and dirty conditions. Inoculated slides were placed inside a SBC into UVSC for 30 s following the manufacturer instructions. In addition, inoculated slides were introduced into a sterile tube containing 10 mL of each disinfectant, 96%, 70% and 60% ethanol or 4%, 2% and 1% chlorhexidine, for 1 h, according to recommended concentrations of use [16].

Subsequent experiments were carried out with the slides inoculated with the six selected microorganisms in clean and dirty conditions. In order to standardize the method, the same exposure times of 30, 60, 90 and 120 s were used with the three disinfectants: UVSC equipment, 70% ethanol and 2% chlorhexidine.

### 2.6. Assessment of the Disinfectant Capacity on Discs of Different Materials

Disinfection capacity evaluation on different materials was performed with a Gram-negative bacterium (*P. aeruginosa* CECT 108), a Gram-positive bacterium (*S. aureus* CECT 435) and a yeast (*C. albicans* ATCC MYA-2876). These pathogens were inoculated on borosilicate, polycarbonate, polyurethane, silicone, Teflon and titanium discs, in clean and dirty conditions. Discs were exposed for 30, 60, 90 and 120 s to the UVC light inside UVSC. Besides, discs under both conditions were also tested with 70% ethanol and 2% chlorhexidine at immersion times of 30, 60, 90 and 120 s.

### 2.7. Colony Counting and Data Analysis

After exposure to UVC inside UVSC and to both disinfectants, slides and discs were aseptically introduced into a sterile tube containing 10 mL of phosphate buffer saline for 5 min with a previous 15 s mechanical agitation to release cells in order to neutralize the effect of disinfectants. After that, aliquots of 0.5 mL were inoculated on plates of TSA and SDA in quadruplicate, being able to detect 5 CFUs in 10 mL; therefore, the detection limit corresponded to 0.5 CFU/mL. After incubation at 37 ± 1 °C for 48 h, colonies per plate were visually and automatically counted using the ChemiDoc XRS System (BioRad, Hercules, CA, USA). Those plates with <330 CFU were used to calculate the number of CFU/mL. According to the standards EN14561 and EN14562, a log_10_ reduction ≥5 was required for considering effective a disinfection method. This log reduction implies the elimination of 99.999% of microbial burden [16,17].

## 3. Results

### 3.1. Assessment of the Disinfectant Capacity on Glassware Slides

Initial assays carried out with exposure times of 30 s in the UVSC equipment showed disinfectant activity, as well as the two disinfectant solution tested at three different concentrations each, applied during 1 h. 

UVSC achieved a reduction of five logarithms in the slides inoculated with *S. aureus* CECT 435 and *P. aeruginosa* CECT 108: from an initial inoculum of 2.4 × 10^9^ CFU/mL of *S. aureus* and 2.3 × 10^9^ CFU/mL of *P. aeruginosa* to 1.5 × 10^2^ and 1.1 × 10^3^ CFU/mL, respectively. The effect of ethanol varied depending on the concentration used. With 96% ethanol, a reduction of five logarithms was achieved (up to 5.5 × 10^2^ in *S. aureus* and 2.2 × 10^2^ CFU/mL in *P. aeruginosa*). Similar results were obtained with 60% ethanol (reduction up to 6 × 10^2^ in *S. aureus* and 2.7 × 10^2^ in *P. aeruginosa*). When 70% ethanol or chlorhexidine were tested, no microbial growth was detected.

**Figure 2:**
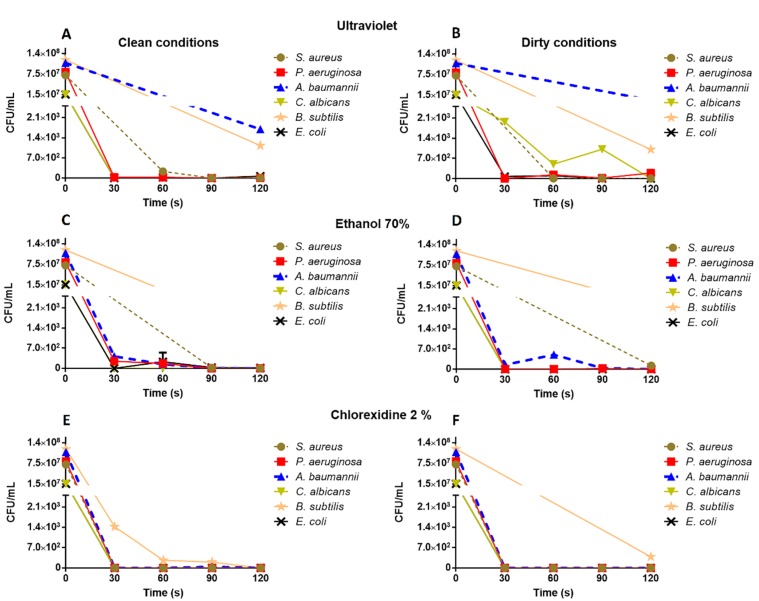
Microbial burden values obtained after the exposure to ultraviolet radiation in UVSC (**A**: clean conditions, **B**: dirty contidions) and the immersion in 70% ethanol (**C**: clean conditions, **D**: dirty contidions) and 2% chlorhexidine (**E**: clean conditions, **F**: dirty contidions). The lines that do not have a term indicate values that are inside the excluded range of the y axis and are not close to the reduction of 5 logarithms established by the standard as a disinfection value.

Based on the results of these first experiments, UVSC, 70% ethanol and 2% chlorhexidine were assayed against *S. aureus*, *P. aeruginosa*, *A. baumannii*, *E. coli*, *B. subtilis* and *C. albicans* with exposure times of 30, 60, 90 and 120 s. Results in both clean and dirty conditions are represented in Figure 2.

UVSC equipment achieved a reduction of more than five logarithms (>99.999%) of *S. aureus*, *P. aeruginosa*, *E. coli*, *B. subtilis* and *C. albicans* burdens at 120 s (Figure 2A,B). However, *A. baumannii* burden was decreased by four logarithms without reaching the threshold considered as disinfection. 

Ethanol, under clean conditions, eliminated the growth of *E. coli* and *C. albicans* completely at 30 s, of *P. aeruginosa* at 90 s and of *S. aureus* and *A. baumannii* at 120 s. Conversely, ethanol did not disinfected slides spiked with *B. subtilis* (Figure 2C). Disinfection was similar under dirty conditions, but total reduction of *S. aureus* burden was not achieved (Figure 2D). 

Chlorhexidine eliminated microbial burden completely at 30 s under both conditions, except for slides contaminated with *B. subtilis* that required 120 s of immersion under clean conditions and did not reach total reduction in dirty conditions (Figure 2E,F).

### 3.2. Assessment of the Disinfectant Capacity on Different Materials

The results of the disinfectant capacity on the discs of different materials inoculated with *P. aeruginosa* CECT 108, *S. aureus* CECT 435 and *C. albicans* ATCC MYA-2876, in both clean and dirty conditions are shown in Table 1, were results obtained at 30 and 120 s exposition times are included.

Disinfection was achieved after exposure of discs inoculated with *P. aeruginosa* to UVC, ethanol and chlorhexidine, in clean conditions. UVC exposure for 30 s reduced four logarithms the microbial burden in borosilicate and Teflon, and up to five logarithms for 120 s exposition in borosilicate, polycarbonate, silicone and titanium. Disinfection of borosilicate was achieved at 30 s, and of polycarbonate and titanium at 120 s in dirty conditions. However, no disinfection was reached in silicone, polyurethane and Teflon discs, although reduction of microbial burden of polyurethane and Teflon discs was higher than four logarithms (99.99%). 

The treatment of discs with ethanol achieved five to seven logarithms reduction in all materials at 120 s in clean conditions, but in dirty conditions, disinfection of polyurethane and silicone was not achieved. Chlorhexidine reduced eight logarithms the microbial burden in all materials studied (Table 1). 

*S. aureus* inoculated discs of borosilicate, silicone, Teflon and titanium were disinfected after 120 s of exposition to UVC. However, polycarbonate and polyurethane discs were not disinfected under clean conditions, nor were silicone discs disinfected under dirty conditions. Similar results were detected when 70% ethanol was used for 120 s, which did not disinfect polycarbonate and Teflon discs in clean conditions or silicone discs in dirty conditions. Effectivity of 2% chlorhexidine was very high, obtaining eight logarithms reduction in all materials (Table 1).

Discs inoculated with *C. albicans* and disinfected with UVSC achieved a reduction of up to four logarithms after 30 s in borosilicate and polycarbonate, and up to 5 to 7 logarithms after 120 s. In polyurethane and silicone, at least a four logarithms reduction was achieved in both conditions, clean and dirty. Fungal burden reduction up to five logarithms was also detected after the immersion in 70% ethanol during 30 s in polycarbonate, polyurethane and titanium in clean conditions, and in all materials at 120 s in both conditions, except for Teflon. Chlorhexidine was very active after 30 s of treatment on all materials and microorganisms (Table 1).

## 4. Discussion

Physical UVC disinfection is a reliable alternative to chemical disinfection due to the increase of chemical-resistant microorganisms and the emission of harmful by-products after chemical treatment. Moreover, UVC disinfection does not generate toxins or volatile organic compounds and does not require storage of hazardous materials. UVC disinfection relies on 250–280 nm wavelength radiation to inactivate pathogens as it penetrates microbial cells, disrupting DNA and affecting reproduction and survival [13,15]. In the current study, we have analyzed the disinfection capability of a portable, easy to use, automated and safe UVC light emitting disinfecting device. The effect observed over a wide variety of microorganisms related to HAIs revealed an appropriate disinfectant capacity at ≤120 s exposure against *P. aeruginosa*, *E. coli*, *S. aureus*, *C. albicans* and *B. subtilis*. Disinfection capacity of this no-touch technology device, considering disinfection as a reduction of 5 logarithms of microbial burden, was similar to 70% ethanol. Ethanol is currently used in protocols for disinfecting different surfaces at medical centers and in handwashing in order to reduce hand colonization of healthcare workers and to diminish pathogen transmission [18,19]; although, 70% ethanol offered higher reductions of microbial burden.

The reduction of the microbial burden of some pathogens was lower with UVC; for instance, a reduction of 3.8 logarithms of *A. baumannii* burden was obtained using UVC. These results agree with those obtained by Rutala et al. [20] that showed a similar reduction with an increase in the time of radiation of 15 min, when performing room disinfection with an UVC device. Ethanol did not disinfect *B. subtilis* contaminated slides and a greater exposure of 120 s to UVC was required to achieve disinfection. Setlow [21] also detected a higher resistance of *B. subtilis* spores to UVC treatment due to the repair system of this bacterium. In addition, Thomas [22] described a certain level of ethanol resistance associated with its ineffectiveness against bacterial spores. In the current study, chlorhexidine exhibited the best disinfectant capacity in all cases, which has also been observed by other authors [13,14]. Koscova et al. [23] observed a 100% reduction of enteric bacteria on mobile phone surfaces, and a similar reduction of *S. aureus*, *Streptococcus* spp., yeast or moulds on computer keyboards. However, it should be underlined that chlorhexidine and ethanol treatments require either immersion or humidification of surfaces, and these processes not always can be carried out with current objects or materials used in medical practice due to possible deterioration of the material or the electronics. These devices could be easily treated using UVSC that also keeps them disinfected for a long time inside a bag. Furthermore, an important clinical consideration is the potential of development of bacterial resistance, tolerance or insusceptibility to chlorhexidine. Kampf [24] reported chlorhexidine resistance by certain bacteria, such as *P. aeruginosa*, *A. baumannii*, *S. aureus*, but not others. There are studies describing an increased presence of the resistance genes *qacA/B* and *smr* in methicillin-resistant *S. aureus* at surgical ICUs [25,26]. However, this increase has not been reported in other study [27].

Disinfection ability of UVC has been demonstrated in studies evaluating room disinfectant capacity of this radiation [28,29]. Yang et al. [28] reported that Hyper Light P3 mobile device killed multidrug-resistant pathogens, such as *P. aeruginosa*, *A. baumannii*, *S. aureus*, *Enterococcus faecium*, *Mycobacterium abscessus* and *Aspergillus fumigatus*, after 5 min irradiation at a distance of 1 m. Furthermore, Umezawa et al. [29] tested other pulsed UVC portable device that disinfected objects inoculated with multidrug-resistant *P. aeruginosa*, *E. coli*, amikacin and ciprofloxacin-resistant *A. baumannii*, methicillin-resistant *S. aureus* and *Bacillus cereus*. The main difference among above mentioned devices and UVSC is that UVSC is designed to disinfect objects and not for room disinfection. These devices for room disinfection can cause harm to health workers if exposed to UVC. This is not the case with UVSC because UVC radiation takes place inside the equipment with the lid tightly closed.

Microorganisms adhere and grow on biomaterials, such as medical and surgical metals, plastics or crystals. The phenotypes of planktonic organisms found in a culture or sessile organisms within biofilms and the effect that disinfectants and antiseptics can have on cleaning them are a very important factors to consider [10,13]. UVSC showed an adequate capacity of disinfection and could be used for disinfecting medical devices, such as stethoscopes, or everyday objects, such as mobile phones or television controls, present at hospital rooms, as well as for inanimate objects outside the hospital setting that can present microbial contamination such as dental prostheses or pacifiers. These objects are composed of different materials in which microorganisms can adhere, survive or even grow, so that their behavior in disinfection may vary. For this reason, further experiments were carried out in the current study inoculating a variety of materials used in the manufacture of those objects, such as borosilicate, polycarbonate, polyurethane, silicone, Teflon and titanium, with *P. aeruginosa*, *S. aureus* and *C. albicans*.

Residual burden for each microorganism after UVC treatment varied with different material studied, probably in association with different porosity, roughness and stability of each material that affect to the initial adhesion of microbial cells to inanimate surfaces [30,31]. Thus, disinfection was more effective in titanium and borosilicate with the lowest hydrophobicity. Previous experiments carried out in our laboratory showed a lower ability of microorganisms to form biofilms on these materials [32]. The materials in which the final counts of CFU/mL were slightly higher were polyurethane and silicone that are very hydrophobic. These results are in accordance with other studies [31,32,33,34] that showed the importance of the hydrophobic effect of the biomaterial surface in the initial adhesion, where bacterial adhesion to the less hydrophobic materials were significantly lower than to the more hydrophobic ones (silicone). Higher roughness seems also to exert some effect in bacterial adhesion [31]. Similarly, the Teflon sample tested in this study shows a very rough surface that promotes bacterial attachment due to the increase in the contact area [33,34]. This could explain the results obtained with *P. aeruginosa* inoculated in Teflon where the reduction in the microbial burden was lower than in other materials. Polycarbonate disinfection was slightly less effective in the case of *S. aureus*; this may be due to the greater adhesion capacity of this microorganism [35].

Overall, UVSC equipment was effective disinfecting slides inoculated with *P. aeruginosa*, *E. coli*, *S. aureus*, *B. subtilis* and *C. albicans* and discs of borosilicate and titanium inoculated with all microorganisms. Although in some cases no disinfection was achieved (a reduction of five logarithms), a notable decrease, ≥99.95%, of microbial burden was obtained. Therefore, UVSC equipment could be a worthy disinfection method to be implemented routinely in hospitals and laboratories at every moment to rapidly disinfect objects in contact with both patients and healthcare personnel, contributing to the control of infection transmission.

## 5. Conclusions

Ultraviolet radiation applied for 120 s using the UVSC equipment was effective in disinfecting slides inoculated with four microorganisms involved in healthcare-associated infections such as *P. aeruginosa*, *E. coli*, *S. aureus* and *C. albicans*. The effect was even greater against spore-forming *B. subtilis*, obtaining an effect similar to that of 70% ethanol and 2% chlorhexidine.

UVSC achieved a high reduction in the microbial burden when treating discs of several materials usually present in objects of medical practice, and/or daily use (borosilicate, polycarbonate, polyurethane, silicone, Teflon and titanium) in both conditions, being the reduction higher than 99.95% in borosilicate, Teflon and titanium, as well as 70% ethanol and 2% chlorhexidine. 

In conclusion, the UVSC equipment is a promising alternative for implementing disinfection protocols in hospitals and other health care settings to inanimate objects that can be used both inside and outside these settings, thus reducing risk of infection transmission.

## Figures and Tables

**Table 1:** Microbial burden values expressed in CFU/mL obtained after exposure of the six materials to 30 and 120 s to the different disinfection methods, UVSC, 70% ethanol and 2% chlorhexidine, in both clean and dirty conditions. Lower detection limit: ≤0.5.

Strain	Initial Inoculum (CFU/mL)	Disinfection Conditions			Materials					
					Borosilicate	Polycarbonate	Polyurethane	Silicone	Teflon	Titanium
***P. aeruginosa* CECT 108**	1.7 × 10^8^	Clean	UVSC	30″	4.6 × 10^3^ ± 41.7	≥	≥	≥	4.2 × 10^3^ ± 141.4	≥
				120″	≤0.5	3.3 × 10^2^ ± 128	5.5 × 10^3^ ± 50.3	1.6 × 10^3^ ± 136.6	6.5 × 10^3^ ± 40	3.0 × 10^1^ ± 38.3
			Ethanol	30″	4.5 × 10^1^ ± 50	0.5 × 10^1^ ± 10	≥	3.0 × 10^2^ ± 31	4.5 × 10^2^ ± 137.9	1.4 × 10^2^ ± 16.3
				120″	≤0.5	0.5 × 10^1^ ± 10	1.5 × 10^3^ ± 244.9	3.0 × 10^1^ ± 11.5	7.5 × 10^1^ ± 34.1	≤0.5
			Chlorhexidine	30″	≤0.5	≤0.5	≤0.5	≤0.5	≤0.5	≤0.5
				120″	≤0.5	≤0.5	≤0.5	≤0.5	≤0.5	≤0.5
		Dirty	UVSC	30″	5.4 × 10^2^ ± 80	3.5 × 10^3^ ± 200.1	≥	≥	≥	5.1 × 10^3^ ± 50.4
				120″	≤0.5	6.0 × 10^1^ ± 95.2	5.8 × 10^3^ ± 81.6	≥	3.8 × 10^3^ ± 141.4	3.3 × 10^2^ ± 40.8
			Ethanol	30″	4.9 × 10^3^ ± 210.2	3.6 × 10^2^ ± 91.4	4.9 × 10^3^ ± 206.1	4.9 × 10^3^ ± 290.6	≥	1.3 × 10^3^ ± 200.0
				120″	0.5 × 10^1^ ± 10	0.5 × 10^1^ ± 10	≥	6.5 × 10^3^ ± 150.2	3.2 × 10^2^ ± 162.4	1.0 × 10^1^ ± 11.5
			Chlorhexidine	30″	≤0.5	≤0.5	2.5 × 10^1^ ± 50	≤0.5	≤0.5	≤0.5
				120″	≤0.5	≤0.5	≤0.5	≤0.5	≤0.5	≤0.5
***S. aureus* CECT 435**	1.2 × 10^8^	Clean	UVSC	30″	≥	≥	≥	≥	≥	≥
				120″	2.8 × 10^2^ ± 78.3	≥	≥	6.0 × 10^2^ ± 66.5	≤0.5	5.1 × 10^2^ ± 91.4
			Ethanol	30″	6.3 × 10^3^ ± 76.7	≥	3.8 × 10^3^ ± 50.3	≥	≥	3.9 × 10^3^ ± 180.3
				120″	≤0.5	≥	1.5 × 10^1^ ± 19.1	5.5 × 10^2^ ± 92.9	2.6 × 10^3^ ± 91.0	≤0.5
			Chlorhexidine	30″	≤0.5	1 × 10^1^ ± 20	≤0.5	≤0.5	≤0.5	≤0.5
				120″	≤0.5	≤0.5	≤0.5	≤0.5	≤0.5	≤0.5
		Dirty	UVSC	30″	≥	≥	≥	≥	≥	≥
				120″	9.5 × 10^2^ ± 17	≥	≥	≥	3.7 × 10^2^ ± 26.7	3.7 × 10^2^ ± 26.7
			Ethanol	30″	≤0.5	≥	≥	≥	≥	≥
				120″	≤0.5	≥	9.8 × 10^2^ ± 42.4	≥	≥	≤0.5
			Chlorhexidine	30″	≤0.5	0.5 × 10^1^ ± 10	4.5 × 10^1^ ± 10	≤0.5	≤0.5	≤0.5
				120″	≤0.5	≤0.5	≤0.5	≤0.5	≤0.5	≤0.5
***C. albicans* MYA-2876**	1.4 × 10^7^	Clean	UVSC	30″	3.5 × 10^2^ ± 131	9.9 × 10^2^ ± 158.7	≥	4.5 × 10^3^ ± 120.7	5.0 × 10^3^ ± 170.0	3.1 × 10^3^ ± 140.4
				120″	≤0.5	1.3 × 10^2^ ± 44.3	2.8 × 10^3^ ± 150.5	5.0 × 10^2^ ± 48.9	2.5 × 10^1^ ± 5.7	0.5 × 10^1^ ± 10
			Ethanol	30″	≥	3.5 × 10^1^ ± 30	≤0.5	2.8 × 10^2^ ± 50	2.8 × 10^3^ ± 228.2	7.5 × 10^1^ ± 19.1
				120″	≤0.5	≤0.5	≤0.5	≤0.5	0.5 × 10^1^ ± 10	≤0.5
			Chlorhexidine	30″	≤0.5	≤0.5	≤0.5	≤0.5	≤0.5	≤0.5
				120″	≤0.5	≤0.5	≤0.5	≤0.5	≤0.5	≤0.5
		Dirty	UVSC	30″	2.0 × 10^1^ ± 9.1	2.0 × 10^2^ ± 82.2	≥	≥	≥	1.3 × 10^3^ ± 98.9
				120″	≤0.5	1.1 × 10^2^ ± 10	3.8 × 10^3^ ± 280.5	5.7 × 10^3^ ± 90.8	1.3 × 10^2^ ± 5	7.0 × 10^1^ ± 25.8
			Ethanol	30″	4.4 × 10^3^ ± 140.0	6.4 × 10^3^ ± 136.6	≥	≥	≥	2.6 × 10^3^ ± 160.7
				120″	≤0.5	≤0.5	≤0.5	3.5 × 10^1^ ± 10	4.1 × 10^2^ ± 131.2	≤0.5
			Chlorhexidine	30″	≤0.5	≤0.5	≤0.5	≤0.5	≤0.5	≤0.5
				120″	≤0.5	≤0.5	≤0.5	≤0.5	≤0.5	≤0.5

Mean log_10_ CFU/plate ± SD; ≥: colony counting ≥ 6.6 × 10^3^ as specified in the standard.
